# Criterion validity of the Short Mood and Feelings Questionnaire and one- and two-item depression screens in young adolescents

**DOI:** 10.1186/1753-2000-4-8

**Published:** 2010-02-09

**Authors:** Isaac C Rhew, Kate Simpson, Melissa Tracy, James Lymp, Elizabeth McCauley, Debby Tsuang, Ann Vander Stoep

**Affiliations:** 1 Social Development Research Group, University of Washington, Seattle, WA, USA; 2 Section of Health Services Research, Baylor College of Medicine, Houston, TX, USA; 3 Department of Epidemiology, University of Michigan, Ann Arbor, MI, USA; 4 Seattle Children's Hospital, Seattle, WA, USA; 5 Department of Psychiatry and Behavioral Sciences, University of Washington, Seattle, WA, USA; 6 Department of Epidemiology, University of Washington, Seattle, WA, USA

## Abstract

**Background:**

The use of short screening questionnaires may be a promising option for identifying children at risk for depression in a community setting. The objective of this study was to assess the validity of the Short Mood and Feelings Questionnaire (SMFQ) and one- and two-item screening instruments for depressive disorders in a school-based sample of young adolescents.

**Methods:**

Participants were 521 sixth-grade students attending public middle schools. Child and parent versions of the SMFQ were administered to evaluate the child's depressive symptoms. The presence of any depressive disorder during the previous month was assessed using the Diagnostic Interview Schedule for Children (DISC) as the criterion standard. First, we assessed the diagnostic accuracy of child, parent, and combined scores of the full 13-item SMFQ by calculating the area under the receiver operating characteristic curve (AUC), sensitivity and specificity. The same approach was then used to evaluate the accuracy of a two-item scale consisting of only depressed mood and anhedonia items, and a single depressed mood item.

**Results:**

The combined child + parent SMFQ score showed the highest accuracy (AUC = 0.86). Diagnostic accuracy was lower for child (AUC = 0.73) and parent (AUC = 0.74) SMFQ versions. Corresponding versions of one- and two-item screens had lower AUC estimates, but the combined versions of the brief screens each still showed moderate accuracy. Furthermore, child and combined versions of the two-item screen demonstrated higher sensitivity (although lower specificity) than either the one-item screen or the full SMFQ.

**Conclusions:**

Under conditions where parents accompany children to screening settings (e.g. primary care), use of a child + parent version of the SMFQ is recommended. However, when parents are not available, and the cost of a false positive result is minimal, then a one- or two-item screen may be useful for initial identification of at-risk youth.

## Background

Although depressive disorders are common in children and adolescents, many depressed youth do not seek or receive either psychiatric evaluation or treatment [1-3]. Without effective treatment, depression can leave children and adolescents with psychological sequelae that increase vulnerability to recurring depressive episodes, impaired occupational functioning, and lowered life satisfaction [4-6]. Accurate identification is an important first step toward providing appropriate intervention for youth with a depressive disorder. Indeed, the U.S. Preventive Services Task Force recently updated their assessment of the appropriateness of screening for depression in adolescents 12 to 17 years-old from "insufficient evidence for or against" in 2002 to recommending screening where systems are established to ensure accurate diagnosis and provision of psychotherapy and follow-up [7]. In a community setting, screening can be challenging given limited access to mental health professionals and the costs and time involved in administering comprehensive assessments such as a structured or semi-structured diagnostic interview [8,9].

Screening questionnaires for depression provide an alternative way to identify at-risk youth, as they can be completed in a brief amount of time and can be administered to large groups of individuals simultaneously. Commonly utilized self-report depressive symptom scales include the 27-item Children's Depression Inventory (CDI), the 30-item Reynolds Adolescent Depression Scale, and the 33-item Mood and Feelings Questionnaire (MFQ) [10-12]. Each takes approximately 10 minutes to administer. An abbreviated, 13-item version of the MFQ, the Short Mood and Feelings Questionnaire (SMFQ), was developed as a brief instrument to evaluate core depressive symptomology in epidemiological studies of children aged 8 to 18 years [13]. The SMFQ takes five minutes or less to complete and can easily be scored on the spot. Parallel versions for parent and child are available. Although one study examined the validity of MFQ in a mixture of children from clinical settings and the community, most studies have been conducted using clinical samples [14-17]. Further, the validity of the SMFQ has been evaluated in only one sample of children recruited from pediatric clinics, one sample of detained adolescents and in one small non-clinical sample of twin pairs [13,18,19].

Furthermore, recent studies on depression in adult samples suggest that screening instruments containing two items that assess depressed mood and anhedonia or just one item assessing mood bear comparable psychometric properties to more lengthy screening measures [20-22]. For example, in a sample of primary care patients, a screen consisting of two questions, one about mood and another about anhedonia, exhibited psychometric properties identical to or better than those of the Zung Depression Scale [21]. While very brief and accurate depression screening tools would be of great value in epidemiological surveys as well as in clinical settings and community-based screening programs, the validity of a very brief one- or two-item screen has not been adequately explored in children or in community samples. A one-item screen used in a national population-based survey to describe trends in depressed mood among adolescents - the Youth Risk Behavior Survey - was found to have moderate test-retest reliability [23,24]. However, the validity of this screen compared to a criterion standard has not been evaluated. Assessments consisting of only one or two items from the SMFQ might good candidates for a very brief screening instrument. Confirmatory factor analyses have observed strong unidimensionality and high internal consistency for the SMFQ in community samples which suggests that one or two items from this scale may be adequate to detect a depressive condition [25].

This current study examines the validity of the SMFQ, as well as that of very brief one- and two-item screens using questions from the SMFQ. The study is conducted in a large, school-based community sample with strong representation of African Americans, Asian American/Pacific Islanders, and European Americans and uses the results of the administration of the Diagnostic Interview Schedule for Children (DISC) depression module to child and parent as the criterion standard. The study compares the sensitivity, specificity, and AUC of three versions of the SMFQ that differ by reporter (child, parent, combined).

Using items from the SMFQ, we also assess the validity of two shorter screens: 1) a two-item scale consisting of the depressed mood and anhedonia items, and 2) a one-item scale consisting only of the depressed mood item. We also address the question of whether there are conditions that might warrant the use of different versions, based on the situation and the accuracy of the specific version of the measure.

## Methods

### Participants

The sample consisted of 521 sixth grade middle school students, aged 11 to 13 years, who participated in the Developmental Pathways Project (DPP), a longitudinal study of co-occurring and non-co-occurring depression and conduct problems. A two-stage sampling approach was employed for DPP. First, a universal mental health screening was carried out with sixth grade students in four consecutive years (2001-2004) at four Seattle-area public schools which were chosen as representative of the Seattle public middle school population [26]. These schools are located in four distinct geographic and demographic areas within Seattle and together have a racial/ethnic distribution that is nearly identical to the total enrolled population of the school district. Students who had a third grade reading comprehension level or higher were eligible to participate. Of the 2,928 eligible students, 2,188 (74.7%) completed the mental health screening which included the MFQ and the Youth Self Report (YSR) [27]. Details of this screening procedure have been described elsewhere [28].

For the second stage of sampling, each year following screening, a random sample of students, stratified by their scores on the MFQ and YSR externalizing scale for conduct problems, was identified for participation in the longitudinal study. Screened students were first assigned to one of four groups based on their screening results: high depressive and high conduct problem score (CO-OCCUR), high depressive and low conduct problem score (DEP), low depressive and high conduct problem score (CP), and low depressive and low conduct problem score (NEITHER). These groups were formed using a cut-off of 0.5 SD above the screening sample mean for the SMFQ and the YSR externalizing scales. Three students who were missing all information from the externalizing module of the YSR were excluded. Students who had been screened, who had at least one parent who could speak English, and who were still residing in the district, were eligible for recruitment into the longitudinal study. A stratified random sample of 807 students was selected for longitudinal follow-up with students scoring high on depressive and/or conduct problem scores sampled according to a ratio of 1:1:1:2 from the four psychopathology screening groups (CO-OCCUR, DEP, CP, and NEITHER, respectively). Because the ratio of these groups was approximately 1:1:1:6 in the general school population, this sampling approach yielded an over-representation of children in the CO-OCCUR, DEP, and CP groups. Oversampling of children with elevated psychopathology scores was carried out to increase the likelihood of observing depressive and conduct disorders over the course of the longitudinal study. Of those selected, 521 students and their parents/guardians (64.6%) consented to participate. Among students who declined participation, there was a greater percentage of Asian American and a smaller percentage of non-Hispanic White children compared to those who enrolled. However, the enrolled and non-enrolled students were similar in gender composition (proportion of males: 52.1% vs. 47.9%; p = 0.49) and mean SMFQ scores at screening (5.9 vs. 6.1, p = 0.68).

Participating students and parents/guardians received an in-home interview administered by two research interviewers who had completed a 16-hour training conducted by DPP investigators. Interviewers were blind to the psychopathology risk group status of the students. The Institutional Review Board of the University of Washington reviewed and approved the study.

### Measures

The data used for this analysis were collected during the baseline interview of the longitudinal study. Students were administered either the 33-item child version of the MFQ (MFQ-C) (n = 483) or the 13-item SMFQ (n = 38), and the child's primary caregiver completed the 34-item parent-version of the MFQ (MFQ-P) to evaluate the child's depressive symptoms over the past two weeks. Lay-administered structured diagnostic interviews were then conducted with each child and parent using the computerized version of the DISC, version four [29]. The parent and child MFQ and the parent and child DISC were administered within a single two-to-three hour period. For both parent and child, the MFQ was always administered before the DISC. Students and parents were interviewed in separate rooms to ensure privacy, and study responses were kept confidential.

#### SMFQ

The 13 items of the MFQ that comprise the SMFQ focus on affective and cognitive symptoms, including one item pertaining to low mood (*I felt miserable or unhappy*) and one item addressing anhedonia (*I didn't enjoy anything at all*) [13]. The informant rates each statement as 2 (*true*), 1 (*sometimes true*), or 0 (*not true*) over the past two weeks, yielding a maximum total score of 26. The developers of the SMFQ found it to have good internal reliability [13]. In addition to total scores for the child (SMFQ-C) and parent (SMFQ-P) versions, we also calculated a combined child and parent score (SMFQ-C+P) by summing the two scores. Daviss et al. found that the summed child and parent MFQ score demonstrated moderate to high criterion validity (.89) for discriminating 7 to 17-year-olds with and without major depressive episodes [14].

#### Brief 1- and 2-item screens

For this study, we extracted the low mood and anhedonia items of the baseline SMFQ to constitute the two-item screen (maximum total score of four). To derive the one-item screen, we used the low mood item alone (maximum total score of two). We selected these two items because they are present on the MFQ as well as other brief depression screening scales such as the PHQ-9 and CDI, and anhedonia and/or depressed mood (or irritability) must be present for a DSM-IV diagnosis [13]. Furthermore, these symptoms show high stability amongst depressed youth [30,31]. Although irritability can be substituted for depressed mood for a depression diagnosis, we elected not to use this item in the brief screen because it is not present in the SMFQ and a number of participants in our study only completed the SMFQ.

#### DISC

The DISC has been commonly used to diagnose depression and other psychiatric disorders in epidemiologic research [32-35]. The DISC has acceptable internal consistency, test-retest reliability, and criterion validity, and the computerized version of the depression module has been shown to have high agreement with physician assessments of depression [36,37]. Interviewers for this study completed 8 hours of classroom trainingand 5 hours of field training before administeringthe fully structuredcomputerized version of the NIMH Diagnostic Interview Schedule for Children (DISC-IV) from one of the project investigators who wascertifiedto train by the Columbia University DISC Development Group. In addition, quality assurance checkswere conducted by project leadership, and feedback was given regarding adherence to study protocol. Interviews were scored by computer, and for this study a positive diagnosis of depression for the previous month was assigned if a child met full symptom criteria for major depressive disorder, dysthymic disorder, or minor depression as specified in the Diagnostic and Statistical Manual of Mental Disorders, fourth edition (DSM-IV) [38]. Although minor depression is not currently a clinical diagnosis per se, its criteria are presented in the DSM-IV as needing further study, and the consequences of this condition are severe as suggested by its association with poor functional outcomes, increased utilization of health services including psychiatric treatment, and a highly increased risk of future major depressive episode [39-42]. Minor depression was defined as the presence of between two to four depressive symptoms for at least two weeks with at least one of the symptoms being loss of interest or pleasure or depressed or irritable mood. In prior studies a one-year time frame has been used to assess criterion validity [13]. We chose to use a past-month diagnosis because it more closely reflects the two-week reference period for the SFMQ. For this study, a combined child-parent diagnosis was ascertained where a positive diagnosis was reported for any one of the three depressive disorders when self- and parent-reported criteria endorsements were combined, such that if a criterion received a positive endorsement by either child or parent, it was considered to be present. Compared to child- or parent-report alone, the combined child-parent DISC diagnosis has shown higher sensitivity and higher concordance with clinician-based assessments [43,44].

### Statistical analysis

Two-component weights were developed and applied to all analyses to account for over-sampling of students who screened high for depression and conduct problems and to make the sample demographically similar to the Seattle public middle school population with respect to gender, race/ethnicity, and educational program status (e.g., regular, gifted, special education, English Language Learner). The first component was a sampling fraction weight that was equivalent to the inverse probability of being enrolled based on the four psychopathology screening groups (i.e. number screened in each category divided by the number enrolled in the longitudinal study in that category). The second component was a post-stratification weight that accounted for differences in gender, race/ethnicity, and educational program status between the screening and longitudinal study samples (i.e. percent of screened students in each gender/racial/school program category divided by weighted percentage enrolled in each category). These two weights were multiplied to produce the final weight for each individual and applied to make the estimates of scale validity more reflective of the screened population.

We compared demographic characteristics and SMFQ scores between those with and without a DISC diagnosis of depressive disorder using χ^2^-and t-tests. The validity of depression scores from the SMFQ-C, the SMFQ-P, and the SMFQ-C+P were assessed against a criterion standard of the combined child-parent DISC diagnosis for the previous month. This method was used to evaluate the full version of the SMFQ, the two-item screen assessing low mood and anhedonia, and the one-item screen assessing low mood only. To examine the validity of each screen *vis-à-vis *a DISC depression diagnosis, sensitivity (the proportion of participants classified as positive by the criterion standard that screens positive) and specificity (the proportion of participants classified as negative by the criterion standard that screens negative) were calculated. Receiver operating characteristic (ROC) curves were generated by plotting the sensitivity against 1-specificity, across a range of SMFQ cutoff values, and the area under the ROC curve (AUC) was calculated to assess the accuracy of each screening method against a DISC depression diagnosis. AUC measures the ability of a screening tool to correctly classify individuals as having a health condition or not. Scores can range from 0.5 to 1.0, where 0.5 indicates an uninformative screen, and 1.0 indicates a perfect screen. In this paper, we interpret an AUC of less than 0.7 to have low diagnostic accuracy, 0.7 to 0.9 to have moderate accuracy, and greater than 0.9 to have high accuracy [45].

We evaluated the sensitivity and specificity of the SMFQ, as well as those of the one- and two-item screens, at the nearest cutoff score where sensitivity and specificity intersected on the ROC curve. This approach has been used in prior methodological studies to select a suitable cut point [19]. In other screening applications, contextual factors such as the clinical implications of false positives or the availability of follow-up resources will influence the decision as to whether to maximize sensitivity or specificity.

χ^2 ^and t-tests were conducted using Stata 10.1 (Stata Corporation, College Station, TX) and weighted sensitivity, specificity, and AUC estimates were calculated using the R statistical package (R Development Core Team, 2009). To estimate 95% confidence intervals for the weighted AUCs, we used a custom program using non-parametric bootstraps (details available upon request).

## Results

Two-hundred seventy-two (52.2%) males and 249 (47.8%) females participated in the study. Of our 521 participants, 43.9% were European American, 25.8% were African American, 25.9% were Asian American, and 4.4% were Native American. The mean age of the sample was 11.5 years (standard deviation (SD) = 0.5). Of the 521 children, 507 (97.3%) had a combined child-parent DISC assessment for depression. For the past month prior to the assessment, eight children met diagnostic criteria for major depression (1.2%, weighted), 16 met criteria for minor depression (2.0%, weighted), and seven met criteria for dysthymic disorder (1.3%, weighted), for a total of 31 (4.6%, weighted) meeting study criteria of any depressive disorder. Of 507 children with a DISC assessment, 499 (98.4%) children completed all items of the SMFQ-C, 490 (96.6%) parents completed all items of the SMFQ-P, and 482 (95.1%) had a child-parent total score calculated. All 507 child-parent pairs with a combined DISC assessment completed both the mood and anhedonia SMFQ items. Data were available from the universal mental health screening from which the study participants were selected, enabling us to compare depression screening scores of the children in the current study who had no missing data and those with some missing data. There were no statistically significant differences in mean SMFQ screening scores between those with and without a DISC child-parent assessment (5.92 vs. 6.29; p = .80), between those with and without a complete SMFQ-C (5.93 vs. 5.75; p = .92), or between those with and without a complete SMFQ-P (5.99 vs. 4.13; p = .13).

Table 1 shows demographic characteristics and SMFQ scores for children with and without a DISC depressive disorder diagnosis. There were no statistically significant differences in sex, race, or age. SMFQ and 1- and 2-item scores were significantly higher in the students with a DISC depressive disorder compared to those without for all reporter versions (p < .001 for all comparisons). The SMFQ showed high internal reliability for both the child and parent version (α = .84 for both). The correlation between the SMFQ-C and SMFQ-P was only moderate (r = .29, p < .001).

**Table 1:** Characteristics of the sample according to DISC depressive disorder diagnosis

	Depressed (N = 31)	Non-depressed (N = 476)
Female, n (%)	14 (45.2)	226 (47.8)
Age, mean years (SD)	11.5 (.6)	11.5 (.6)
Race, n (%)		
Native American	2 (6.5)	17 (3.6)
Black	9 (29.0)	137 (28.8)
Asian American	1 (3.2)	91 (19.1)
White	19 (61.3)	231 (48.5)
SMFQ version, mean (SD)		
Child*	8.2 (6.1)	3.8 (3.6)
Parent*	7.3 (5.8)	3.1 (3.2)
Combined child and parent*	15.9 (7.4)	6.9 (5.5)
2-item version, mean (SD)		
Child*	1.3 (1.0)	.7 (.8)
Parent*	1.6 (.9)	.8 (.8)
Combined*	2.9 (1.4)	1.5 (1.2)
1-item version, mean (SD)		
Child*	.90 (.60)	.51 (.55)
Parent*	1.03 (.60)	.60 (.58)
Combined*	1.94 (.81)	1.11 (.88)

* p-value < .001

### Validity of the total SMFQ score

**Figure 1:**
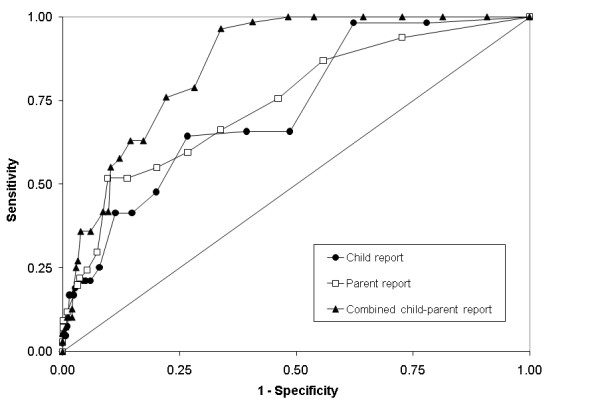
**Receiver operating characteristic curves for the full SMFQ against DISC depression diagnosis via child report, parent report, and combined child and parent report**.

Figure 1 shows the ROC curves for the child, parent, and combined SMFQ. All three versions showed moderate diagnostic accuracy for DISC depression diagnosis (Table 2). The SMFQ-C and SMFQ-P were very similar with regard to validity estimates. The SMFQ-C showed an AUC of 0.73 (95% CI: 0.63-0.84). At a cut point of four, where sensitivity and specificity most closely intersected, we observed a sensitivity of 0.66, and specificity of 0.61. The SMFQ-P had an AUC of 0.74 (95% CI: 0.62-0.85), and again, a score of four or more emerged as the cut point where sensitivity and specificity intersected, corresponding to 0.66 sensitivity, 0.66 specificity. Of the SMFQ versions, the SMFQ-C+P displayed the highest AUC (0.86; 95% CI: 0.81-0.91). Graphs of sensitivity and specificity intersected at a cut point of 10 with 0.76 sensitivity, and 0.78 specificity.

**Table 2:** Area under the Receiver Operating Characteristic Curve (AUC), sensitivity, and specificity of SMFQ depression screening methods against the DISC diagnosis of depressive disorder based on combined child and parent report.

	Child Report	Parent Report	Child + Parent Report
**Total SMFQ Score**			
N	499	490	482
Number of cases	31	29	29
AUC (95% confidence interval)	0.73 (0.63-0.84)	0.74 (0.62-0.85)	0.86 (0.81-0.91)
Cut point	**4**	**4**	**10**
Sensitivity	0.66	0.66	0.76
Specificity	0.61	0.66	0.78
**SMFQ Mood and Anhedonia Questions**			
N	507	506	506
Number of cases	31	31	31
AUC (95% confidence interval)	0.67 (0.56-0.78)	0.74 (0.63-0.84)	0.78 (0.68-0.88)
Cut point	**1**	**1**	**2**
Sensitivity	0.81	0.86	0.77
Specificity	0.50	0.46	0.58
**SMFQ Mood Question alone**			
N	507	506	506
Number of cases	31	31	31
AUC (95% confidence interval)	0.66 (0.54-0.78)	0.65 (0.53-0.76)	0.71 (0.58-0.83)
Cut point	**1**	**1**	**2**
Sensitivity	0.72	0.76	0.65
Specificity	0.57	0.49	0.68

### Validity of the two-item screen

**Figure 2:**
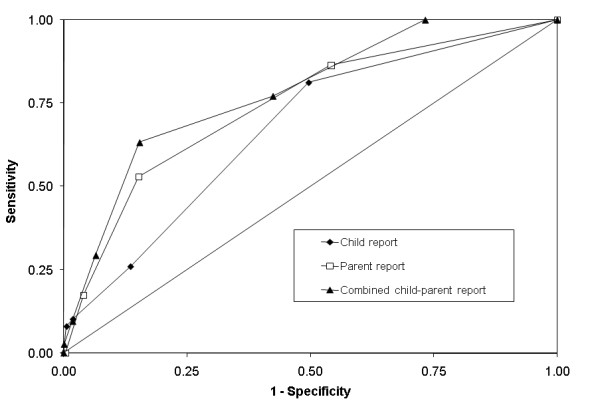
**Receiver operating curves for the two-item screen against DISC depression diagnosis via child report, parent report, and combined child and parent report**.

ROC curves for each version of the two- item screens are presented in Figure 2. We observed low diagnostic accuracy for the child-version (AUC = 0.67, 95% CI: 0.56-0.78) (Table 2). Sensitivity and specificity curves intersected at a cut point of one, yielding a sensitivity of 0.81, and specificity of 0.50. The parent- and combined-versions showed moderate diagnostic accuracy. The parent version had an AUC of 0.74 (95% CI: 0.63-0.84). A cut point of one yielded sensitivity of 0.86 and 0.46 specificity. Combining child and parent two-item screen scores, we observed an AUC of 0.78 (95% CI: 0.68-0.88), with 0.77 sensitivity, and 0.58 specificity at a score of two.

### Validity of the one-item screen

**Figure 3:**
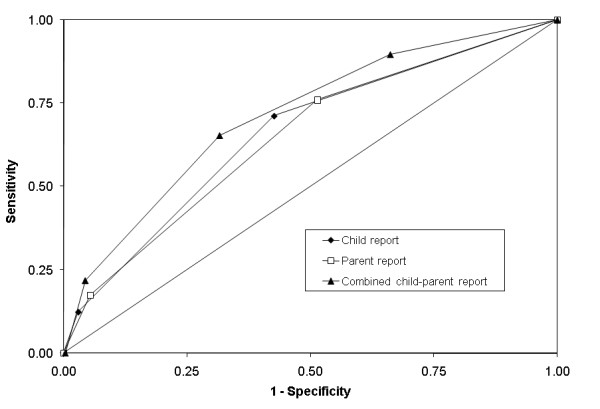
**Receiver operating curves for the one-item screen against DISC depression diagnosis via child report, parent report, and combined child and parent report**.

Figure 3 shows the ROC curves for the different reporter versions of the one mood-item screen. Both the child- and parent-report demonstrated low diagnostic accuracy. By child report, response to the item "I felt miserable or unhappy" had an AUC of 0.66 (95% CI: 0.54-0.78). Sensitivity and specificity graphs intersected at a cutoff score of one, where sensitivity was 0.72, and specificity 0.57. By parent report, the mood item demonstrated an AUC = 0.65 (95% CI: 0.53-0.76). Sensitivity and specificity again intersected at a cut point of one, with 0.76 sensitivity and 0.49 specificity. Combining child and parent scores on the mood item resulted in moderate diagnostic accuracy (AUC = 0.71, 95% CI: 0.58-83). Sensitivity and specificity graphs intersected at a cut point of two, with sensitivity of 0.65 and specificity of 0.68.

## Discussion

As expected, the accuracy of the SMFQ varied depending upon the version that was used. The most labor-intensive screening approach produced the best approximation of a valid depression diagnosis. The combined child + parent SMFQ yielded an AUC that approached 0.9 while the easier-to-administer one- and two-item versions demonstrated lower accuracy with diminished specificity. However, factors other than accuracy may inform decisions about the choice of a screening tool. Our study results indicate that under screening conditions where both a parent and child are available to complete a five-minute questionnaire, administering the 13-item SMFQ to both reporters would yield information with the highest sensitivity and specificity. On the other hand, there may be conditions under which only the child is available, and time is limited. Under these circumstances, if screen sensitivity were the primary concern and the cost of yielding a high number of false positives was not too great, then administering the one or two-item screen to children only may be warranted.

Considering the accuracy of these screening tools in light of context is illustrated using a hypothetical sample of 500 children that reflects a typical school or other community setting (Table 3). First, the expected cross-tabulation of true versus screened diagnoses using the SMFQ-C+P as a screen was compared to the distribution yielded when using the SMFQ-C as a screen. Next, the yield of the one- and two-item child report versions were compared. Arguably, sensitivity is acceptably high for three of these four depression screening tools, but differences in specificity are dramatic. Due to low specificity, attempts to estimate the prevalence of depression or screen children on the basis of SMFQ and one- and two-item screen scores set at "optimal" cut points (as determined in this study by convergence of highest sensitivity and specificity) would yield markedly inflated results or many false positives.

**Table 3:** Results from hypothetical screening program with 500 adolescent participants, prevalence of depression = 6/100.

	Combined 13-item SMFQ			Child 13-item SMFQ	
	True +	True -		True +	True -
Screen +	23	103	Screen +	20	183
Screen -	7	367	Screen -	10	287
					
	Child 2-item			Child 1-item	
	True +	True -		True +	True -
Screen +	24	235	Screen +	22	202
Screen -	6	235	Screen -	8	268

Our findings suggest similar accuracy of the child and parent versions of the SMFQ which is in contrast with an earlier study conducted by Thapar and McGuffin [19]. In their community sample of twins that used the Child and Adolescent Psychiatric Assessment (CAPA) semi-structured interview as a criterion standard, the authors found that the SMFQ-C had an AUC of 0.72, sensitivity of 0.75, and specificity of 0.74, whereas the SMFQ-P fared substantially better showing high accuracy with an AUC of 0.90 and sensitivity and specificity of 0.86 and 0.87, respectively. The study sample, however, evaluated children over a wider age range (8 to 16 years). Further, despite administering both child- and parent-versions of the CAPA, Thapar and McGuffin only used the parent-version as their criterion standard. Because child and parent reports of depressive symptoms often do not show good agreement, it may not be surprising that a parent-report screen would show improvement over a child-report screen when compared against a diagnosis based on an interview with the parent, only [46].

To expand on the issue of parent-child agreement, the reliability and accuracy of reporter versions may vary by age. It is commonly accepted that adolescents are more accurate reporters of internalizing symptoms than their parents [47]. However, prior to adolescence, there is concern about whether children can fully comprehend and respond reliability to questions about mood and feelings [48]. Thus, for our sample consisting of sixth grade students (children making the transition to adolescence) the similar validity estimates for child and parent reports that we observed may be in line with this notion.

Consistent with the study conducted by Angold et al. as well as previous studies examining the validity of the full MFQ, we found that of the three SMFQ versions (child, parent, or combined) the SMFQ-C+P scores performed the best [13,14]. However, our SMFQ-C validity was lower than that reported by Angold et al., who found a sensitivity of 0.60 and a specificity of 0.85 at a cutoff of eight. Our most acceptable cutoff was four, and at this lower cut point our observed sensitivity was similar to that of Angold's group, but our specificity was much worse (0.61). There were notable differences between the current study and the Angold study that may contribute to the contrasting findings. First, children in the Angold study were recruited from pediatric clinics where a higher prevalence of illness would be expected, while participants in our study were public middle school students. Second, Angold et al. studied a sample of children with a wider and younger age range, 6 to 11 years. Finally, the relevant period for the criterion standard in their study was 1-year while we used a DISC diagnosis based on symptoms present during the previous one month.

For comparison, we conducted a post-hoc analysis also examining the accuracy of the full 33-item MFQ among the somewhat smaller sample of children who were administered the full MFQ. Interestingly, we found that the MFQ yielded comparable (and, in the case of the child report, even somewhat lower) AUC estimates than their corresponding reporter versions. We observed an AUC of 0.70 for the child, 0.77 for the parent, and 0.85 for the child+parent versions. It is possible that the additional items on the MFQ do not significantly improve diagnostic accuracy for depression beyond those found on the 13-item SMFQ. Because of the little or no loss of accuracy and its reduced size compared to the full MFQ, the SMFQ may be a more desirable choice for screening purposes.

In contrast with studies in adult populations, the present study found that the psychometric properties of the one- and two-item screens are not as desirable as those of the full SMFQ [20-22]. For example, in the one-item screen, 32% of children who do not have a depressive disorder would screen positive by combined child-parent report, versus 22% in the full SMFQ-C+P. While the difference in AUCs between the three different screening methods could not be directly tested due to overlapping items, a noticeable decline in AUC was observed from the full 13-item instrument to the brief one- and two-item screens. Despite this decline, the possible utility of very brief screens cannot be discounted. The two-item screen still showed moderate accuracy for the parent- and combined-versions, and the one-item combined-version also showed moderate accuracy. Further studies using other types of very brief screens with more specific language about duration and severity of the symptom may be useful.

Examination of screens with additional items may be useful. Again, we selected items from the SMFQ *a priori *based on criteria necessary to establish a depression diagnosis and other research suggesting the stability of the items in depressed youth throughout childhood [30,31]. There is some research to suggest that cognitive features may discriminate well for a latent depression construct [49]. Future research could examine whether addition of one or two of such cognitive features to a brief screen could improve accuracy *vis a vis *depression diagnosis.

This study has several limitations. Because of the restricted age range (sixth graders ages 10 to 13 years), findings from this young adolescent sample may have limited generalizability to children in earlier or later stages of childhood and adolescence. It should also be noted that this study sample is on the younger age range of the spectrum for the recommendation for screening for depression by the US Preventive Services Task Force because there is little evidence to suggest that standard treatments are effective for children under 12 years. All sensitivity and specificity estimates are based on the same data that were used to derive the optimal cut point and are likely biased upward. Validation of these cut points on an independent population would be useful. Another limitation was the relatively low level of participation (65% of those randomly selected) and the differing response rates by race (i.e. lower percentage of Asian American students consenting). Although sampling weights were applied to account for differences in race as well as other demographic characteristics, it is possible that those who enrolled were not representative of those who did not. This may further limit external validity. Also, prior research suggests that participants in adolescent studies of mental health involving both child and parent report might be of higher SES than non-participants, which may affect the period prevalence of depression in the sample [32,50-52]. However, because we had initial screening scores for both participants and non-participants, we were able to compare the SMFQ scores between groups. The mean SMFQ score in participants was not significantly different than that of non-participants (p = 0.7), which suggests that non-participants were likely comparable to participants in terms of depression status. There is no indication that more impaired children were excluded. Furthermore, the school context as the screening location needs to be considered when evaluating the accuracy of the SMFQ. In clinical settings, such as primary care clinics or certainly in mental health centers, where the prevalence of depression in the population is higher, the predictive value of a positive screen will improve. Finally, despite apparent differences in accuracy among the versions of the SMFQ that were examined, most confidence intervals for estimates of the AUC overlapped, such that it is difficult to make definitive between-version distinctions in validity. Still, the AUC estimates are reflective of what we would expect to observe across screening versions.

## Conclusions

In this school-based community sample, we found that the SMFQ shows reasonable psychometric properties for identifying children in early adolescence with a depressive disorder. However, unlike findings in adult samples, one- and two-item screens did not bear properties comparable to those of the 13-item screening instrument. Other very brief tools incorporating more specific language about timing and severity of functional impairment or including only a few additional items may prove more suitable. Development of accurate screening measures for adolescent populations is an important first step in addressing depression as a public health problem in our communities. Where appropriate systems are in place for accurate diagnosis, appropriate treatment (i.e. psychotherapy) and follow-up, the SMFQ may be a feasible and useful screening instrument in these settings because of its relative administrative ease, as well as its accuracy.

## Competing interests

The authors declare that they have no competing interests.

## Authors' contributions

KS, IR and AV contributed to the conceptualization of the study. IR, KS, MT, and JL were involved with data analyses. AV and EM oversaw the collection of data. All authors contributed to the writing of the manuscript, and all read and approved the final manuscript.
